# Methods of miRNA delivery and possibilities of their application in neuro-oncology

**DOI:** 10.1016/j.ncrna.2023.10.002

**Published:** 2023-10-07

**Authors:** Ilgiz Gareev, Ozal Beylerli, Rasim Tamrazov, Tatiana Ilyasova, Alina Shumadalova, Weijie Du, Baofeng Yang

**Affiliations:** a Department of Pharmacology (The State-Province Key Laboratories of Biomedicine Pharmaceutics of China, Key Laboratory of Cardiovascular Research, Ministry of Education), College of Pharmacy, 150067, Harbin Medical University, Harbin, China; b Translational Medicine Research and Cooperation Center of Northern China, Heilongjiang Academy of Medical Sciences, Harbin, 150081, PR China; c Central Research Laboratory, Bashkir State Medical University, Ufa, Republic of Bashkortostan, 3 Lenin street, 450008, Russia; d Department of Internal Diseases, Bashkir State Medical University, Ufa, Republic of Bashkortostan, 3 Lenin street, 450008, Russia; e Department of General Chemistry, Bashkir State Medical University, Ufa, Republic of Bashkortostan, 3 Lenin street, 450008, Russia; f Department of Oncology, Radiology and Radiotherapy, Tyumen State Medical University, 54 Odesskaya Street, 625023, Tyumen, Russia

**Keywords:** miRNA, Brain tumors, Delivery system, Therapy, Vectors, Blood-brain barrier

## Abstract

In the current phase of medical progress, practical neuro-oncology faces critical challenges. These include the quest for and development of innovative methodological approaches, as well as the enhancement of conventional therapies to boost their efficacy in treating brain tumors, especially the malignant varieties. Recent strides in molecular and cellular biology, molecular genetics, and immunology have charted the primary research pathways in the development of new anti-cancer medications, with a particular focus on microRNA (miRNA)-based therapy. MiRNAs possess the ability to function as suppressors of tumor growth while also having the potential to act as oncogenes. MiRNAs wield control over numerous processes within the human body, encompassing tumor growth, proliferation, invasion, metastasis, apoptosis, angiogenesis, and immune responses. A significant impediment to enhancing the efficacy of brain tumor treatment lies in the unresolved challenge of traversing the blood-brain barrier (BBB) and blood-tumor barrier (BTB) to deliver therapeutic agents directly to the tumor tissue. Presently, there is a worldwide effort to conduct intricate research and design endeavors aimed at creating miRNA-based dosage forms and delivery systems that can effectively target various structures within the central nervous system (CNS). MiRNA-based therapy stands out as one of the most promising domains in neuro-oncology. Hence, the development of efficient and safe methods for delivering miRNA agents to the specific target cells within brain tumors is of paramount importance. In this study, we will delve into recent findings regarding various methods for delivering miRNA agents to brain tumor cells. We will explore the advantages and disadvantages of different delivery systems and consider some clinical aspects of miRNA-based therapy for brain tumors.

## 1 Introduction

Brain tumors, constituting 85–90 % of all central nervous system (CNS) tumors, represent a significant medical and social challenge within contemporary oncology. Despite the widespread adoption of advanced technologies and improvements in diagnostic and treatment methods, the mortality rate for malignant brain tumors like glioblastoma, anaplastic meningioma, medulloblastoma, or metastatic tumors remains stubbornly high worldwide [1]. Hence, there's a pressing need to explore new unconventional therapeutic approaches. MicroRNAs (miRNAs), short non-coding RNAs (ncRNAs), serve as epigenetic regulators, influencing gene expression by interacting with the 3′- untranslated regions (3′ - UTRs) of mRNA targets. MiRNAs have emerged as pivotal players in the development and progression of brain tumors [1,2]. They can either suppress tumor growth or act as oncogenes, exerting a substantial influence on processes like proliferation, differentiation, tumor metabolism, epithelial-mesenchymal transition (EMT), angiogenesis, metastasis, and drug resistance across various brain tumor types. Consequently, the use of miRNA-based therapy presents new avenues for the prevention and treatment of this group of pathologies.

Leveraging miRNA-based therapy to modulate the expression of tumor-related genes holds great promise for both fundamental scientific research and clinical applications [3]. In the realm of modern neuro-oncology, a significant challenge lies in the low specificity of drugs, including miRNA agents, and the presence of the blood-brain barrier (BBB) and blood–tumor barrier (BTB). Addressing these challenges entails the development of systems tailored for the targeted transport of miRNA agents. MiRNAs equipped with delivery systems offer several advantages: 1) enhanced cellular penetration; 2) improved pharmacokinetics; 3) the ability to surmount cellular membranes and the BBB; 4) provision of essential biocompatibility and safeguards against premature degradation; 5) facilitated targeted transport and controlled release of miRNA agents, among others [4,5]. Additionally, a crucial question concerns the method of delivering miRNA agents to tumor tissue, specifically, which route is safer and more effective for treating brain tumors. Hence, when investigating miRNA-mediated therapy for brain tumors, it becomes imperative to consider potential delivery systems and routes of administration to achieve the desired therapeutic outcome. This study will explore various miRNA agent delivery systems, scrutinizing their advantages, drawbacks, and potential prospects for clinical implementation.

## 2 Problems of effective drug delivery and therapy of brain tumors

**Fig. 1:**
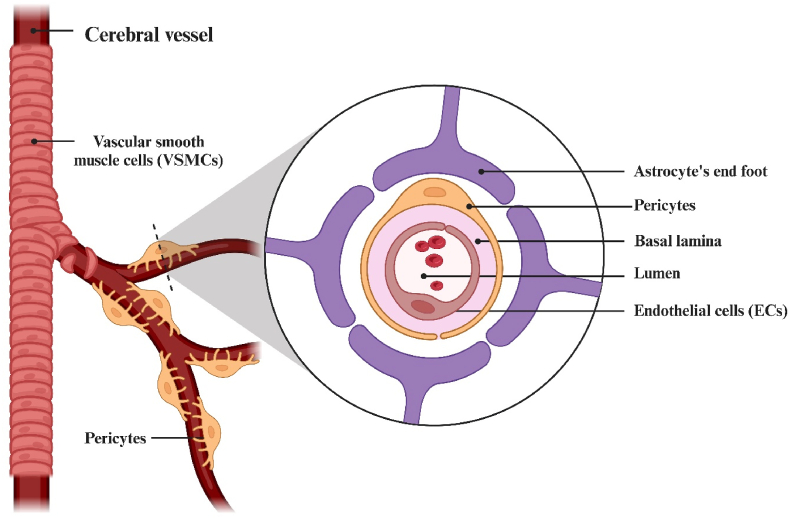
Schematic illustration of the blood-brain barrier (BBB). The existence of the BBB is a necessary and most important condition for the normal functioning of the central nervous system (CNS).

Despite the efficacy of miRNA-based therapy in regulating oncogene expression, there are several structural and biophysical obstacles to delivering miRNA agents to their intended target cells. At the structural level, the BBB is composed of tightly interconnected endothelial cells (ECs) covered with glycocalyx on the outer membrane surface. Additionally, there are pericytes, astrocytes, and elements of the extracellular matrix. The conjunction of the basement membranes of endothelial cells, the end feet of astrocytes, and pericytes forms a neurovascular unit responsible for regulating the BBB's permeability to cells, including immune cells (Fig. 1) [6].

The main proteins that ensure adhesion of ECs and the formation of tight junctions are Claudin-5/24 [7]. On the surface of the cell membranes of the brain capillary network, aquaporin-4/9 (AQP-4/9) are mainly expressed [7]. A decrease in the expression of various AQP-4 isoforms was noted in brain lesions with malignant forms of brain tumors (e.g., glioblastoma), which can affect the integrity of the BBB and contribute to the development of peritumoral edema [8]. Brain tumor cells secrete several substances that violate the integrity of the BBB and its selective permeability, the endothelium changes its charge to positive, blood cells that have a negative charge activate the processes of aggregation and adhesion, closing the site of damage [9]. In brain tumors, microglia can be activated in M1 or M2 phenotypes, respectively, damaging or protecting the integrity of the BBB. Pro-inflammatory cytokines (tumor necrosis factor-alpha (TNFα), interleukin-1β (IL-1β) and interleukin-6 (IL-6)) produced by activated microglia induce rearrangement and changes in the expression of tight junction proteins zonula occluden-1 (ZO-1), which leads to pathological permeability of the BBB [7]. Another reason for the change in permeability is an increase in the expression of cell adhesion proteins (selectin or intercellular adhesion molecule 1 (ICAM-1)), which facilitate the migration of immune cells to the area of inflammation through paracellular mechanisms [10]. In brain tumors, the connection between ECs and pericytes is disrupted. Pericytes contain a large amount of the actin protein involved in cell contraction, they can change the lumen of capillaries and locally regulate blood pressure [7]. BBB disruption is considered as the main diagnostic feature of malignant gliomas, meningiomas, and tumor metastases in the brain, which are detected by contrast-enhanced magnetic resonance imaging (MRI) and computed tomography (CT) [7].

**Fig. 2:**
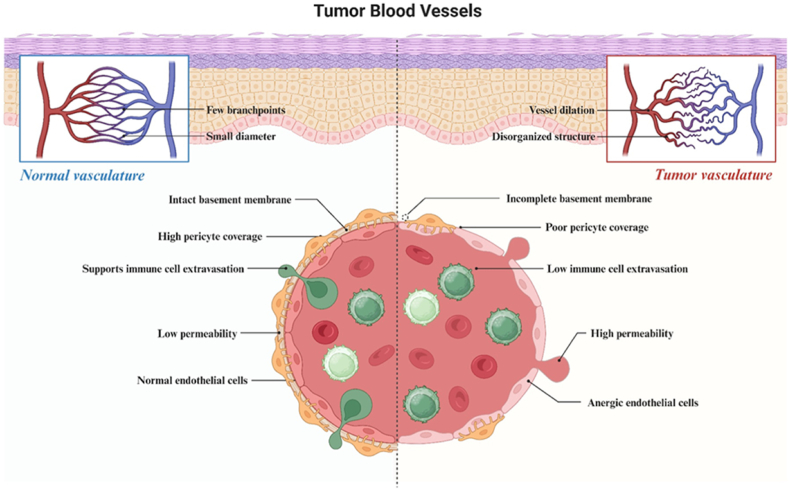
**Comparative characteristics of the structure of the blood-brain barrier (BBB) in normal and blood–tumor barrier (BTB) is in brain tumors.** BTB is generally considered « leakier » than BBB. BTB is characterized by an aberrant distribution of pericytes, and loss of astrocyte end foots and neuronal connections. T cell subpopulations and peripheral monocytes are found in brain tumors, indicating permeability to circulating immune cells. In addition, junctional proteins are reduced in endothelial cells (ECs) of BTB, and the intratumoral vasculature never fully restores normal BBB in brain metastases. Although BTB is "defective", it retains important aspects of the BBB, including the expression of active efflux transporters in ECs and tumor cells.

It is known that in malignant forms of brain tumors extravasation of macromolecules is significantly increased, which is associated with the phenomenon of increased vascular permeability and retention of macromolecules - enhanced permeability and retention effect (EPR effect) [11]. It is known that in a tumor in the zone of actively growing vessels, the BBB cannot be fully formed and BTB is formed. Atypical blood vessels of the tumor have an inferior structure, which causes circulatory pathology: occlusions, embolisms, thromboses, hemorrhages, which prevent the entry of medicinal agents into the tumor tissue during their systemic administration. In addition, when pericytes are absent, BTB is unable to block some neuro- and vasotoxic serum proteins, increasing inflammation in the tumor microenvironment (Fig. 2) [12,13].

Numerous molecules, including therapeutic agents, encounter difficulties in crossing the BBB due to their rapid removal from ECs by both active (ATP-binding cassette transporters (ABC transporters)) and passive (major facilitator superfamily (MFS)) transmembrane proteins. Notably, these proteins, such as P-glycoprotein (P-gp), multidrug resistance protein (MRP), and breast cancer resistance protein (BCRP), are often overexpressed in tumors [14,15].

In patients with malignant gliomas, atypical meningiomas, and brain metastases, BBB dysfunction is a common observation. Tumor growth is associated with decreased outflow of cerebrospinal fluid (CSF) and interstitial fluid, leading to peritumoral (focal) edema and increased BBB permeability to chemotherapy drugs, including miRNA agents. However, evidence suggests that one of the primary reasons for the limited effectiveness of systemic antitumor therapy in malignant brain tumors is the presence of areas with an intact BBB, even within regions of infiltrative tumor cell growth [16]. It can be hypothesized that the permeability of the BBB varies in different tumor zones and the peritumoral area, influenced by factors like the microenvironment and tumor growth stage. The characteristics of the BTB further contribute to creating specific conditions, such as hypoxia and acidosis, which play a crucial role in chemoresistance by disrupting normal processes like angiogenesis, apoptosis, DNA repair, oxidative stress response, immune surveillance, and the activity of multidrug resistance-associated genes [17].

**Fig. 3:**
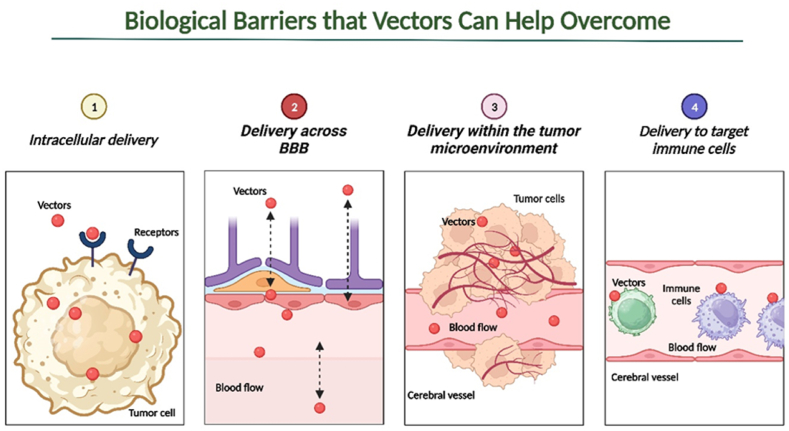
**Schematic illustration of major biological barriers to drugs in the treatment of brain tumors.** It has been shown that with the help of various modifications, as a vector delivery system, it is possible to overcome biological tumor barriers with the effective use of antitumor agents, including microRNAs (miRNAs) agents.

Cell membranes form a vital barrier essential for maintaining cell integrity and normal function. Microviscosity of cell membranes ranks among the critical biophysical parameters of a cell, as alterations can lead to significant disruptions in morphology and physiology. Changes in membrane lipid metabolism are observed in various tumor types [18,19]. Cholesterol synthesis is impaired in tumor cells, with metastatic brain tumors, for instance, exhibiting elevated cholesterol levels. High membrane cholesterol content has been linked to increased drug resistance, possibly due to membrane sealing caused by reduced lipid bilayer voids. Additionally, higher cholesterol levels are associated with an increased number of lipid rafts, domains involved in cell processes such as proliferation, differentiation, apoptosis, migration, and malignant transformation, uncontrolled growth, invasiveness, and metastasis [[20], [21], [22], [23]]. Increased membrane viscosity results in an abundance of proteins such as integrins, adhesins, CD44, and CD24 receptors, which play roles in tumor progression, invasion, and are localized in lipid rafts [24]. Nonetheless, promising outcomes from preclinical investigations suggest that these obstacles can be surmounted through enhancements in carriers and chemical modifications of miRNAs. Vector delivery is particularly pertinent for transporting miRNA agents, as it aids in their traversal of barriers, circumvents the host's immune system, and promotes their more precise accumulation within brain tumor cells (Fig. 3).

## 3 MiRNA-based therapy

To artificially elevate the expression level of a specific miRNA in a target cell, synthetic copies of this molecule, known as miRNA mimics, are introduced (replacement therapy). These mimics can either be mature molecules, which are direct replicas of miRNAs with the same binding properties as the original, or their precursor genes. Using mature miRNA mimics is a more convenient and expedited method of intervention [3]. For example, the transfection of miR-339-5p mimic in vitro demonstrated the potential to suppress the proliferation, invasiveness, migration, and survival of glioblastoma cells [25].

However, when using miRNA mimics to enhance the repression of protein synthesis, it's crucial to remember that active miRNA function relies on the formation of the ribonucleoprotein complex known as the RNA-induced silencing complex (RISC) [26]. It has been observed that introducing individual miRNA molecules into a cell typically encounters a limited pool of proteins. To form a RISC complex, these introduced miRNAs must compete with the endogenous miRNAs within the cell for the protein components of the RISC complex, potentially depleting their reserves [27]. Consequently, this may not only result in a limited impact on the translation of the target mRNA but also an increase in the translation of other proteins due to the disruption of the regulatory functions of the cell's native miRNAs. Studies have even suggested that introducing a miRNA duplex consisting of both leading and passenger strands is more likely to yield an active miRNA molecule compared to introducing a mature single-stranded molecule [27]. The potential use of primary miRNA (pri-miRNA) or precursor miRNA (pre-miRNA) in the study of brain tumors has also been explored [28]. In summary, the use of miRNA mimics offers extensive possibilities for substitution therapy in combatting brain tumors.

Another approach involves the use of antisense inhibitors, which are RNA oligonucleotides complementary to the target miRNA [3]. When they bind, a strong duplex form, preventing the miRNA from binding to the target mRNA and, consequently, lifting the translation inhibition. Natural regulators of miRNA activity in the body include competitive endogenous RNA (ceRNA), such as long non-coding RNAs (lncRNAs) and circular RNAs (cirRNAs) [29]. These molecules contain miRNA binding sites within their nucleotide sequences and can act as "sponges," essentially assuming the role of miRNA and thus releasing the blockade on target mRNA [30]. For instance, the use of the lncRNA HCG11 resulted in the complete inactivation of oncogenic miR-144, leading to increased apoptosis, decreased proliferative activity, and enhanced resistance to chemotherapy in glioblastoma cells [31].

Exogenous inhibitors also demonstrate effective suppression of target miRNA activity. However, in this case, delivering the molecule to the cell through the membrane and maintaining its stability in the cytoplasm become essential, as an unprotected exogenous RNA molecule can be swiftly degraded by RNases. These challenges are partially addressed by the latest generations of miRNA inhibitors based on locked nucleic acid (LNA)-modified oligonucleotides. These oligomers, 12–14 nucleotides in length, contain a methylene bridge in some nucleotides between 2′-O and 4′-C of the ribose ring. As a result, LNA molecules are more resistant to the action of endonucleases, form stronger duplexes with the target RNA or DNA, penetrate the cell membrane more easily due to their small size, and exhibit minimal toxicity in in vivo experiments [32,33]. These attributes make LNA inhibitors promising candidates for the development of drugs aimed at suppressing the activity of targeted miRNAs in the treatment of brain tumors.

## 4 Vector delivery of miRNA agents

Delivering miRNA agents into a living organism remains a challenging endeavor and is an active area of research and development. Several hurdles complicate this process. Firstly, free RNA molecules lacking protection or modification are susceptible to degradation by nucleases and can be excreted by the kidneys and liver or retained in non-target organs. Secondly, various tissues/structures or barriers, such as the BBB, connective tissues, and the tumor microenvironment, often appear along the path to the tumor cells within the body, posing significant delivery challenges. Thirdly, foreign RNA molecules can trigger an immune response and lead to undesirable side effects. Fourthly, even in cell cultures, where introducing molecules into the nutrient medium might suffice, penetrating the cell necessitates overcoming the cell membrane barrier. Finally, once inside the cell, miRNA may be sequestered within endosomes or engage in nonspecific interactions with non-complementary or partially complementary RNA molecules [[34], [35], [36]].

**Fig. 4:**
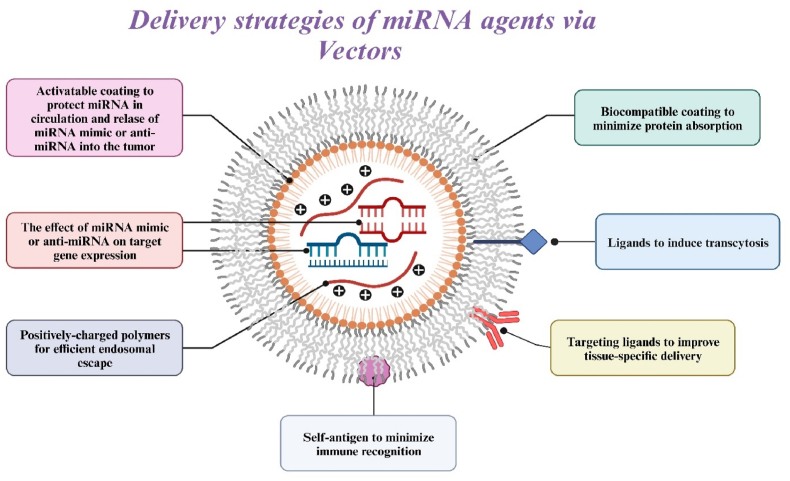
**Strategies to overcome problems with the efficiency, specificity, and safety of delivering miRNA agents to the body to inhibit oncogenes or activate oncosuppressor genes.** The efficiency of the interaction of exogenous microRNAs (miRNAs) (mimics or antagomiRs) with endogenous (tumor) microRNAs and mRNA targets can be increased by increasing the bioavailability for target loci. Approaches to improve efficiency: 1) prevention of enzymatic degradation through stable encapsulation; 2) immunity evasion due to biocompatible coating and self-antigens; 3) increased extravasation due to ligands that induce transcytosis of the vascular endothelium; and 4) enhancement of intracellular and nuclear penetration of miRNAs through cationic polymers. Specificity can be improved by decorating the delivery vehicle with target ligands and designing delivery vehicles that respond to external or tissue-specific signals. Approaches to improve safety: 1) implementation of mechanisms of local retention or systemic inhibition; and 2) the use of biocompatible materials to minimize local inflammation.

Currently, various delivery systems for transporting miRNA agents to brain tumor cells are under development [37]. One of these is the vector delivery system, which encompasses both viral and non-viral delivery systems for miRNA agents [37]. There is existing evidence that demonstrates some degree of safety and efficacy in delivering miRNA mimics or antagomirs to brain tumor target cells. Moreover, modification with vector systems circumvents several limitations associated with the delivery of miRNA agents, including susceptibility to nuclease degradation, rapid clearance from the bloodstream, immunotoxicity, and low permeability to tumor cells (Fig. 4). However, it's important to note that each vector system has its own set of advantages and disadvantages, which must be carefully considered for the effective utilization of miRNA-based therapy in clinical practice in the near future.

## 5 Viral delivery systems for miRNA agents

**Fig. 5:**
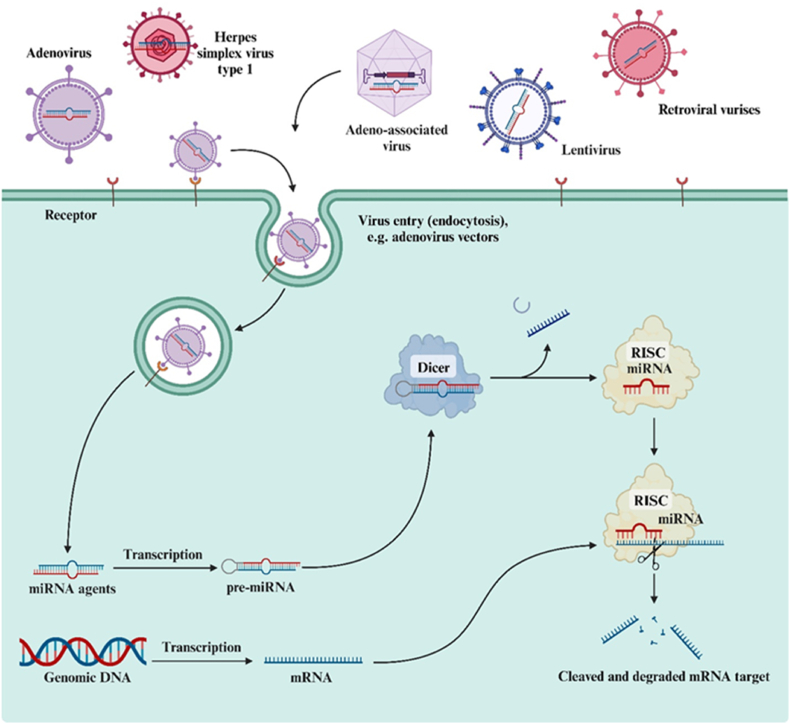
**Viral delivery systems for microRNA (miRNA) agents.** Various types of viral vectors are considered, such as retroviral, adeno-associated, and lentiviral vector systems, vector systems based on adenoviruses, and herpes simplex virus type 1.

Presently, viral vectors serve as a widely employed tool for delivering miRNAs into cells [37]. There exists a wide array of viral vectors, each possessing its own set of advantages and disadvantages (Table 1) [[38], [39], [40]]. Considerable efforts are currently directed towards enhancing the safety and efficiency of miRNA delivery into cells using viral vectors. Additionally, there is a focus on ensuring long-term and tissue-specific expression of the introduced miRNA agent (Fig. 5). Viral vectors hold the potential to be one of the methods for delivering miRNAs in the treatment of brain tumors. In fundamental neuro-oncology, viral vectors have found application as efficient systems for delivering miRNAs to tumor cells both in vitro and in vivo (Table 2) [[41], [42], [43], [44], [45], [46], [47], [48], [49]].

**Table 1:** Comparative characterization of most used viral vectors with their advantages and disadvantages for potential use in neuro-oncology.

Virus	Genome	Size, nm	Titer, transducing units in ml^−1^	Capacity, thousand base pairs	Target cells	Duration of miRNA expression	Integration with recipient DNA	Transduction efficiency	Immunogenicity
LVs	RNA	100	10^6^–10^9^	8	Dividing cells	↑	+	↑	↓
AVs	Double stranded DNA	80–120	10^9^–10^13^	20	Dividing and non-dividing cells	↓	–	↑	↑
AAVs	Single stranded DNA	20–30	10^9^–10^13^	4,5–5,0	Dividing and non-dividing cells	↑	+-	↑	↓
HSVs	Double stranded DNA	120–300	10^8^–10^11^	30–50 (up to 150 for amplicons)	Non-dividing cells	↓	–	↓	↓

**Abbreviations:** LVs, Lentiviruses; AVs, Adenoviruses; AAVs, Adeno-associated viruses; HSVs, Herpes simplex viruses. **Note:** +, Possible; -, No possible; +-, Sometimes possible; ↑, High; ↓, Low.

**Table 2:** Viral delivery system of microRNA (miRNA) agents in brain tumors treatment studies in vitro and in vivo.

Tumor type	Type of virus	miRNA	Agent	Characteristics of carriers	Gene target	Effect of miRNA	Reference
GB	LVs	miR-28-5p	Mimic	Efficient delivery	FOXO1	Promotes tumor spheres formation, cell viability, and proliferation	[41]
GB	LVs	miR-218	Mimic	Efficient delivery	Bmi1 and HK2	Reduces of proliferation, migration, and invasion	[42]
MB	LVs	miR-218	Mimic	Efficient delivery	SH3GL1	Suppresses tumor cell growth, migration, and invasion	[43]
MB	LVs	miR-22	Mimic	Efficient delivery	PAPST1	Reduces cell proliferation and induces apoptosis	[44]
MB	AVs	miR-199b-5p	Mimic	Efficient delivery	HES1	Reduces cell proliferation and tumor growth	[45]
MB	AVs	miR-34a	Mimic	Efficient delivery. No signs of toxicity or morbidity	Cyclin D1, cMyc and CDK4	Reduces cell proliferation, and induces apoptosis and neural differentiation	[46]
GB and AS	AVs	miR-124/TIKI2	Mimic	Efficient delivery and TIKI2 protected normal cells from toxicity	WNT signaling pathway	Reduces gliomageneis	[47]
GB	oHSV	miR-124	Mimic	Efficient delivery and does not replicate in mouse brain or cause disease	EGFR	Reduces gliomageneis	[48]
GB	BVs	miR-10b	Mimic	Efficient delivery	HOXD10, NOTCH1, TP53, and PAX6	Reduces tumor growth, invasion, and angiogenesis	[49]

**Abbreviations:** AS, Astrocytoma; GB, Glioblastoma; MB, Medulloblastoma; BVs, Baculoviruses; LVs, Lentiviruses; AVs, Adenoviruses; oHSV, oncolytic herpes simplex virus; FOXO1, Forkhead Box O1; Bmi1, Polycomb complex protein; HK2, Hexokinase 2; SH3GL1, SH3 Domain Containing GRB2 Like 1, Endophilin A2; PAPST1, Adenosine 3′-phospho 5′-phosphosulfate transporter 1; HES1, Hes Family BHLH Transcription Factor 1; CDK4, Cyclin-dependent kinase 4; EGFR, Epidermal growth factor receptor; HOXD10, Homeobox D10; NOTCH1, Neurogenic locus notch homolog protein 1; TP53, Tumor protein p53; PAX6, Paired Box 6.

### 5.1 Retroviral vector systems

The Retroviridae family comprises seven main genera, and their representatives are of significant interest as a platform for creating new viral vectors for delivering miRNA agents. Most of the research in this field has primarily focused on gamma-retroviruses and lentiviruses such as human immunodeficiency virus 1 (HIV-1) [50]. The limited efficiency of transducing non-dividing cells by gamma-retroviral vectors restricts their utility for miRNA transfer into stem cells [51]. In contrast, lentiviral vectors (LVs) are capable of transducing non-dividing and slowly dividing cells, greatly expanding their potential applications for miRNA delivery to various tissues and organs, including the CNS [52]. Despite HIV-1 serving as the foundation for developing lentiviral vectors, genetic engineering techniques enable the creation of vectors that are safe for potential patients. Specifically, genes responsible for virus replication, packaging, and export from the cell are removed from the viral genome. Consequently, lentiviral vectors maintain their functional activity while losing their replicative capability. However, it is essential to consider certain safety aspects when using lentiviral vectors. Integration into the host cell genome occurs randomly, which can potentially trigger insertional oncogenesis. Nonetheless, the risk of host cell degeneration can be minimized by optimizing the vector's composition and structure [53,54].

For example, Li et al. demonstrated the successful transfer of miR-519a mimic into glioblastoma cells using a lentiviral vector (LV-miR-519a mimic) and applied it to a subcutaneous xenograft model [55]. Initially, the authors observed reduced expression of miR-519a in chemoresistant glioblastoma tissues and temozolomide (TMZ)-resistant cells, suggesting an association between low miR-519a levels and TMZ resistance. Subsequently, their results indicated that miR-519a enhanced chemosensitivity in glioblastoma cells, primarily through TMZ-induced autophagy and apoptosis, by targeting the signal transducer and activator of transcription 3 (STAT3)/B-cell lymphoma 2 (Bcl-2) signaling pathway.

In another study, Zhen et al. demonstrated the transfer of miR-524-5p mimic using lentiviral-based vectors into pituitary-derived folliculostellate (PDFS) cells isolated from nonfunctional pituitary adenomas (NFAs) [56]. The study confirmed the tumor-suppressor function of miR-524-5p in PDFS cells by showing inhibited cell proliferation, migration, invasion, and clonogenicity in vitro. Furthermore, in vivo experiments exhibited the tumor growth inhibitory effects of miR-524-5p, supporting the hypothesis that miR-524-5p possesses tumor-suppressing properties. The study also suggested that PBF is a putative target gene of miR-524-5p.

### 5.2 Adenoviruses and adeno-associated viral vectors

The process of creating recombinant adenoviral vectors shares similarities with the creation of lentiviral vectors. Adenoviruses themselves are unable to replicate effectively due to the replacement of the E1 gene, which is crucial for replication. However, these recombinant vectors can efficiently propagate in cells expressing the E1 gene product [57]. Recombinant adenoviral vectors can achieve very high expression of cloned genes but only for a short duration (typically 5–10 days) due to the immune response generated by the recipient organism [58]. To address this limitation, the second generation of adenoviral vectors was developed. In these vectors, genes responsible for virus replication, in addition to the E1 gene, were deleted, leaving only the elements that determine the start and end of the genome and the viral packaging sequence. Such vectors are capable of sustaining miRNA expression for a longer period. Adenoviruses have the ability to infect a wide range of cells, both dividing and non-dividing [59]. For example, it has been found that recombinant adenovirus can infect glioblastoma stem cells following direct intracranial injection [60].

Yao et al. demonstrated that oncolytic adenoviruses (OA) containing multiple miRNA response elements (MREs) (miR-124, miR-128, miR-146b, and miR-218) suppressed the growth of glioma xenografts without causing toxicity to normal tissues [61]. Specifically, they found no significant difference in serum levels of alanine transaminase (ALT) between mice treated with phosphate-buffered saline (PBS) and those treated with OA-4MREs, indicating no hepatotoxicity induced by adenovirus treatment.

Recombinant adeno-associated viruses (AAVs) are among the most promising vectors for miRNA delivery due to their non-pathogenic nature, low host immunogenicity, ability to target most cell types and tissues, high transduction efficiency, and long-term expression capabilities [62]. However, one challenge with AAVs is that the human population continually encounters various serotypes of adeno-associated viruses, leading to the production of antibodies that neutralize AAVs. To address this issue, modifications to the immunogenic epitopes of AAVs or the use of rarer capsid serotypes have been proposed [63]. There are a total of 8 different serotypes known in AAVs, with AAV2 being the most extensively studied and widely used as a delivery vector for miRNA agents [64]. Bhere et al. engineered AAV-miR-7 and stem cells (SC) releasing secretable (S)-tumor necrosis factor apoptosis-inducing ligand (TRAIL) to regulate miR-7 expression. They used pharmacological inhibition of caspases and genetic loss of function to investigate the impact of forced miR-7 expression on death receptor (DR) pathways in glioblastoma with established TRAIL resistance and in patient-derived primary glioblastoma stem cell (GSC) lines [65]. The results of their experiments demonstrated that a single administration of AAV-miR-7 significantly reduced tumor volumes, upregulated DR5, and enabled SC-delivered S-TRAIL to eradicate glioblastoma xenografts generated from TRAIL-resistant patient-derived GSCs, ultimately improving the survival of mice. This study highlighted the effective delivery of miR-7 to the glioblastoma microenvironment using AAV vectors.

### 5.3 Other types of viral vectors

Herpes simplex virus 1 (HSV-1) viral vectors have a simpler design compared to adenovirus vectors. HSV-1 itself comprises around 80 genes, with one of them (IE3) commonly replaced when creating a vector [66]. It's also possible to exclude other genes, allowing for vector expansion or cloning several genes of interest. However, HSV-1 vectors have some drawbacks, including transient expression of cloned genes, toxicity to target cells, low transduction efficiency, and the ability to infect only non-dividing cells. HSV-1 is neurotropic and effective for studying retrograde and anterograde transport in the CNS and can be introduced in a benign latent state. HSV-1 vectors have a large genetic capacity and can provide long-term miRNA expression. However, their primary disadvantages are cell toxicity and low transduction efficiency [67,68].

Nevertheless, some studies have demonstrated the oncolytic HSV-mediated expression of miRNAs and effective and specific silencing of oncogenes in brain tumor cells both in vitro and in vivo [48,69]. For instance, Otani et al. successfully transferred miR-H16 using oncolytic herpes simplex virus (oHSV) in glioblastomas both in vitro and in vivo [69]. They found that HSV-1 induces the activity of the neurogenic locus notch homolog protein (NOTCH) signaling pathway through HSV-1 encoding miR-H16, which is overexpressed during productive infection.

For the therapy of brain tumors, some viral constructs traditionally used in other areas have also been adapted. For example, based on baculovirus vectors widely used in genetic engineering, vectors for in vivo transduction have been created [70]. Current developments related to baculovirus vectors for miRNA-based therapy in vivo and ex vivo are focused on tissue engineering, including osteogenesis, tumor therapy, and vaccine development [49,71,72].

## 6 Non-viral delivery systems for miRNA agents

**Fig. 6:**
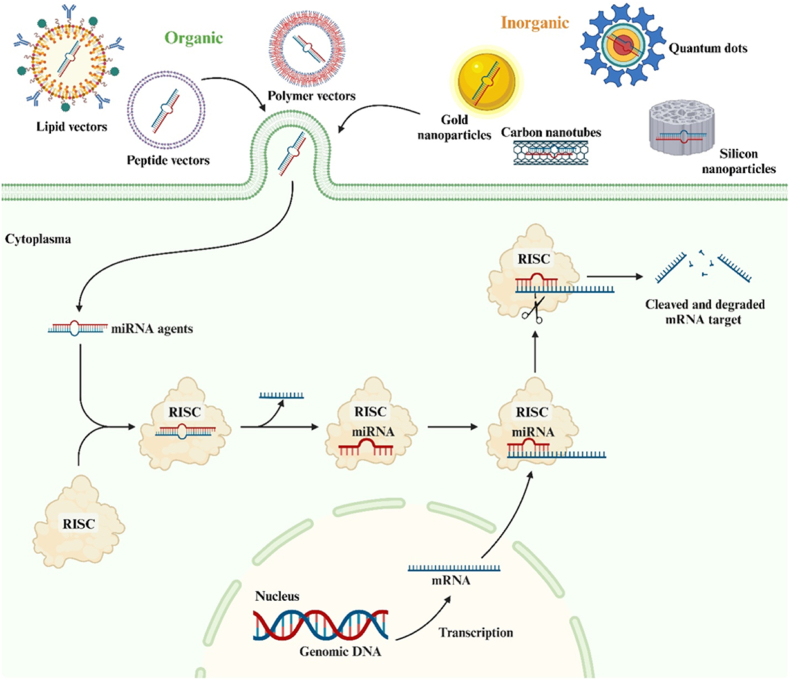
**Non-viral delivery systems for microRNA (miRNA) agents.** Non-viral vectors are represented by organic, synthetic, and inorganic compounds.

In contrast to viral vectors, the category of non-viral delivery systems for miRNA agents is highly diverse. Various types of complexes and nanoparticles (NPs) with sizes ranging from 1 to 1000 nm are currently utilized for the transport of miRNA. These include polymers, cationic peptides, liposomes, quantum dots, carbon nanotubes, silicone nanoparticles, and other inorganic nanoparticles (Fig. 6) [73,74]. Furthermore, this vector system has demonstrated its effectiveness in delivering miRNA agents for brain tumor therapy, both in vitro and in vivo (Table 3) [[75], [76], [77], [78], [79], [80], [81], [82], [83], [84], [85], [86]].

**Table 3:** Non-viral delivery system of microRNA (miRNA) agents in brain tumors treatment studies in vitro and in vivo.

Tumor type	Carrier	miRNA	Agent	Characteristics of carriers	Gene target	Effect of miRNA	Reference
GB	SNA-Liposome-ApoE	miR-92b	Mimic	Inert and nontoxic. Accumulation in tumor tissue and can notably escape lysosome/late endosomes	–	Reduces tumor cell viability	[75]
AS	Lipofectamine2000	miR-323-5p	Mimic	Accumulation in tumor tissue	IGF-1R	Enhances the tumor cell apoptosis	[76]
GB	CTX-coupled SNALPs	miR-21	Ani-miRNA	The loss of host immunogenicity. BBB crossing and accumulation in tumor tissue	RhoB	Enhances the tumor cell apoptosis and decreases tumor cell proliferation and angiogenesis	[77]
GB	Cationic liposomes	miR34a and miR21	Mimic and ani-miRNA	Accumulation in tumor tissue	EGFR	Reduces tumor cell viability, proliferation, and invasion	[78]
GB	PAMAM	miR-7	Mimic	Higher transfection efficiency and longer duration of action compared with liposome delivery	EGFR	Suppresses tumor cell proliferation and tumor growth	[79]
GB	Liposome	miR-20a	Mimic	Accumulation in tumor tissue	–	Enhances the tumor cell proliferation and the percentage of S-phase cells.	[80]
GB	FA/PAMAM	miR-7	Mimic	Makes a longer time for the drug action and accumulation in tumor tissue	PCNA, MMP-2, MMP-9, EGFR and AKT-2	Reduces tumor cell viability, proliferation, and invasion	[81]
GB	CPPs, R8	miR-21	Ani-miRNA	Inert and nontoxic. Accumulation in tumor tissue and can notably escape lysosome/late endosomes	PDCD4 and SERPINB5	Tumor cell migration inhibition	[82]
GB	Tachyplesin	miR-210	Ani-miRNA	Electrostatically bind to miRNA to form a stable complex and protect the nucleic acid from degradation. BBB crossing and accumulation in tumor tissue	NeuroD2 and HIF3A	Inhibits proliferation, migration and spheroid formation ability and induces apoptosis and sensitivity to temozolomide	[83]
GB	NFL-TBS.40-63 peptide	miR-21, miR-221 and miR-100	Ani-miRNA	Can enter specifically into glioblastoma cells by endocytosis (mitochondria specific)	PTEN, FGFR3 and NAIP	Reduces tumor cell viability, induces microtubule destruction, and inhibits cell proliferation	[84]
GB	PLGA nanoparticles	miR21 and miR-10b	Ani-miRNA	PLGA nanoparticles as efficient carriers of antisense miRNAs offer both localized high concentrations of intracellular antisense miRNAs and gradual and sustained release of encapsulated molecules over a long period	PTEN, PDCD4, and HOXD10	Antiproliferative and cytotoxic effects, induces cell cycle arrest at G2/M phase	[85]
GB	PU-PEI	miR-145	Mimic	Accumulation in tumor tissue	Sox2 and Oct4	Enhances chemoradiosensitivity and reduction of CSC-like properties	[86]

**Abbreviations:** AS, Astrocytoma; GB, Glioblastoma; SNA-Liposome-ApoE, Creating spherical nucleic acids-liposome-apolipoprotein E; PU-PEI, Polyurethane-short branch polyethylenimine; TMZ, Temozolomide; PAMAM, Poly(amido amine); FA, Folic acid; CPPs, Cell penetrating peptides; PLGA, Poly(lactic-co-glycolic acid); CSC, Cancer stem cells; IGF-1R, Insulin like growth factor1 receptor; RhoB, Ras homolog gene family, member B; EGFR, Epidermal growth factor receptor; PCNA, Proliferating cell nuclear antigen; MMP-2, Matrix metalloproteinases 2; MMP-9, Matrix metalloproteinases 9; AKT-2, V-akt murine thymoma viral oncogene homolog 2; PDCD4, Programmed cell death 4; SERPINB5, Serpin family B member 5; NeuroD2, Neuronal differentiation 2; HIF3A, Hypoxia-inducible factor 3 alpha; PTEN, Phosphatase and tensin homolog; FGFR3, Fibroblast growth factor receptor 3; NAIP, Neuronal apoptosis inhibitory protein; PDCD4, Programmed cell death 4; HOXD10, Homeobox D10; Sox2, SRY-Box transcription factor 2; Oct4, Octamer-binding transcription factor 4.

### 6.1 Lipid vectors

Lipid vectors, or liposomes, are highly organized lipid structures consisting of one or more concentric closed bilayers of phospholipids with hydrophobic heads and hydrophilic tails, creating an inner aqueous core. Liposomes have proven to be effective for delivering water-soluble substances placed within their hydrophilic core [87]. Their widespread use in miRNA delivery is attributed to their optimal size (around 100 nm), excellent biocompatibility, and ease of preparation and utilization [88]. For instance, the neutral lipid 1,2-oleoyl-sn-glycero-3-phosphocholine (DOPC) can encapsulate up to 70 % of miRNAs by mixing solutions of the two components [89]. Liposomes can also be prepared from other neutral lipids such as dioleoylphosphatidylethanolamine (DOPE), 1,2-distearoyl-sn-glycero-3-phosphocholine (DSPC), phosphatidylcholine (PC), and more [88,90]. Liposomes were the first nanoparticles approved for clinical use, such as polyethylene glycol (PEG)-coated liposomes containing doxorubicin [91].

Complexes formed by cationic lipids and nucleic acids are referred to as lipoplexes [92]. The key advantage of cationic lipids lies in their ability to interact passively with negatively charged miRNAs and the cell's plasma membrane, which greatly facilitates internalization. However, it should be noted that cationic liposomes may exhibit higher toxicity than neutral ones, possess a shorter serum half-life (partly due to uptake by the reticuloendothelial system), and increased immunogenicity (due to uptake by macrophages) [93,94]. One commonly used cationic lipid is 1,2-dioleoyl-3-trimethylammonium-propane (DOTAP), known for its ability to maintain a positive charge across physiological pH values and effectively concentrate anionic miRNA [95]. Biontech, for example, employed a combination of DOTAP and a fusion-assisting lipid coating in their cancer therapy platform to create lipid complexes. Moreover, the ratio of cationic lipids in DOTAP can be adjusted to selectively target antigen-presenting spleen cells for miRNA delivery. Several ongoing preclinical trials have shown promising therapeutic outcomes using these complexes in the treatment of tumors, including brain tumors [96,97].

For instance, Wang et al. conducted research on the therapeutic potential of miR-7 in human glioblastoma in vivo. MiR-7, known as a tumor suppressor, plays a crucial role in regulating cell proliferation, apoptosis, and migration. They employed the DOTAP system to deliver miRNA and assessed the anti-tumor effects of miR-7. The systematic delivery of the miR-7/DOTAP complex to subcutaneous glioma xenografts resulted in significant growth inhibition of the primary tumor (approximately 40 % reduction in tumor volume and weight) and metastatic areas (60 % reduction in lung metastases and 80 % reduction in lymph node metastases). These findings provide compelling evidence that miR-7 can effectively inhibit the growth and metastasis of glioma xenografts in vivo, suggesting its clinical feasibility for glioblastoma therapy using the DOTAP system [98].

### 6.2 Peptide vectors

Peptides can also serve as efficient delivery systems for interfering RNA, and a particular category of cationic peptides known as cell-penetrating peptides (CPPs) have demonstrated the ability to transport various macromolecules, including miRNAs, into tumor cells and other target cells [99]. There are currently two primary approaches for utilizing CPPs as miRNA carriers in target cells, including tumor cells. The first approach involves forming a covalent bond between CPPs and miRNAs. This covalent bond is typically established through a disulfide or, less commonly, thioether linkage, which is cleaved within the cell cytoplasm. It's important to note that this strategy may sometimes lead to reduced miRNA efficacy due to incomplete dissociation of the CPP-miRNA complex.

The second approach relies on the formation of CPP complexes with miRNAs through electrostatic interactions. In this method, positively charged CPPs bind to negatively charged miRNAs, resulting in the formation of a highly stable complex that effectively protects miRNAs from degradation by nucleases found in blood serum [100,101]. For example, a study by Jana et al. successfully demonstrated that Tachyplesin can act as a CPP with efficient miRNA delivery capabilities [102]. Tachyplesin is a protective peptide derived from horseshoe crab species known for its antimicrobial and anticancer activity. These peptides have an amphipathic β-hairpin structure, carry a high positive charge, and differ by only one or two amino acid residues. The study revealed that Tachyplesin peptides were effective in delivering anti-miR-210 to glioblastoma cells. The cyclization of the main peptide chain improved stability in human serum and reduced toxicity. Additionally, these peptides demonstrated a strong binding affinity to lipids, appropriate orientation within the cell membrane, and the ability to disrupt the lipid bilayers of tumor cells. However, it's worth noting that this approach carries the risk of neutralizing the positive charge of CPPs during electrostatic interactions with miRNAs, rendering them incapable of binding to the cell's plasma membrane and entering the cell as a CPP-miRNA complex [103,104].

### 6.3 Polymer vectors

Polymers offer significant advantages for miRNA delivery due to their structural flexibility, allowing for easy adjustment of the physicochemical characteristics of the delivery system. Factors such as molecular weight, charge density, solubility, and hydrophobicity can be tailored to specific test conditions. For instance, altering the ratio of polymer to miRNA enables control over the charge neutralization degree of the complex [105]. Additionally, the introduction of various chemical groups can modify polymer properties, giving them new functionalities. Both natural and synthetic polymers are utilized to create polyplex systems for in vivo miRNA delivery [88,106].

Poly(lactic-co-glycolic acid) (PLGA) complexes are widely employed as carriers for miRNA transport. They offer several advantages, including small particle size, low cytotoxicity, and prolonged circulation in the bloodstream. PLGA-miRNA complexes can be prepared in two ways: by incorporating miRNA into the complex core or by adsorbing miRNA onto the surface of modified cationic PLGA nanoparticles through electrostatic interactions. PLGA effectively shields miRNAs from degradation by serum nucleases and facilitates sustained release of the transported substance [107,108].

Polyethyleneimine (PEI) stands out as one of the most efficient miRNA delivery vehicles, primarily due to its excellent endocytosis and endosomolytic activity. High molecular weight PEI (25 kDa) is commonly employed for miRNA transport due to its strong binding capacity with miRNA and effective protection against enzymatic cleavage. However, its clinical application is hampered by high cytotoxicity and limited biodegradability. Low molecular weight PEI (<2 kDa) is less toxic but less efficient in miRNA delivery. It is believed that PEI and similar cationic polymers enhance tumor cell membrane permeability by creating transient nanoholes and may exert cytotoxic effects due to membrane destabilization. The degree of branching in the polymer structure also impacts PEI efficiency and toxicity [108,109].

Polymeric nanoparticles like PLGA and PEI are extensively used as miRNA carriers in glioblastoma therapy [110]. For instance, PLGA nanoparticles have been employed for the delivery of anti-miR-10b and anti-miR-21 to glioblastoma cells both in vitro and in vivo [111,112]. These nanoparticles effectively protect encapsulated miRNAs from degradation by serum nucleases and provide sustained release over an extended period. In vivo experiments using targeted PLGA-PEG nanoparticles encapsulating anti-miR-21 and anti-miR-10b have shown significant antitumor effects in glioblastoma models [113,114]. These findings suggest the potential of polymeric nanoparticles for miRNA-based glioblastoma therapy.

### 6.4 Inorganic nanoparticles

Inorganic nanomaterials or nanoparticles, such as carbon nanotubes, quantum dots, and gold nanoparticles, offer alternative approaches for miRNA delivery. These nanoparticles differ from organic ones in terms of structure, size, physical and chemical properties, and can be easily functionalized despite their low molecular weight [115].

Quantum dots (QDs), colloidal semiconductor nanoparticles, are commonly used as fluorescent probes due to their unique physicochemical properties, which overcome the limitations of fluorescent proteins and organic dyes [116]. QDs exhibit a wide excitation band, allowing excitation of nanocrystals of different colors with a single radiation source, and they produce narrow symmetrical fluorescence peaks with high photostability. These characteristics make QDs effective delivery vehicles for therapeutic miRNAs. For instance, QDs have been utilized for simultaneous imaging and delivery of miRNAs to selectively suppress the expression of the epidermal growth factor III receptor gene (EGFRvIII) in U87 cells [117]. However, the clinical use of QDs is limited due to their high cytotoxicity, primarily because they often contain toxic elements like cadmium (Cd), selenium (Se), or tellurium (Te). As a result, QDs are currently mainly used for in vitro studies. Recently, new types of QDs (I-III-VI2), such as AgInS2, CuInS2, and ZnS·AgInS2, have been developed, demonstrating lower cytotoxicity and the potential for multifunctional use in imaging and miRNA delivery to glioblastoma cells [118,119].

Carbon nanotubes (CNTs) are cylindrical layers of graphene, with single-walled CNTs consisting of a single graphene layer and multi-layer CNTs containing several concentric single-walled nanotubes. While single-walled nanotubes have a diameter of no more than 0.4 nm, multi-layer ones can reach about 100 nm in diameter. The length of these structures varies from hundreds of nanometers to several tens of micrometers [120]. CNTs possess a unique graphene layer that can be readily modified with various biomolecules. Complexes of CNTs with miRNAs can be formed through covalent or non-covalent bonds. Importantly, CNTs are non-toxic to cells and can pass through the cell membrane in an endocytosis-independent manner without compromising its integrity [121].

Gold nanoparticles are well-suited for miRNA transport due to their unique chemical and physical properties. They are inert and non-toxic, with sizes ranging from 1 to 150 nm, allowing particles with a size limit of 15–20 nm to penetrate the blood-brain barrier (BBB). Gold nanoparticles also exhibit anti-angiogenic effects, inhibiting vascular formation and suppressing the proliferation of vascular endothelial cells induced by vascular endothelial growth factor 165 (VEGF165) [122]. These properties make them suitable for delivering miRNAs to glioblastoma tumor cells. For example, brain-targeted gold-liposomal nanoparticles have been designed to effectively deliver anti-miR-92b to glioblastoma cells. These nanoparticles, known as spherical nucleic acids (SNA) - gold liposome-apolipoprotein E (ApoE), increased the internalization of oligonucleotide miRNA inhibitors (OMIs) into glioblastoma cells, reduced miR-92b expression, lowered glioblastoma cell viability, and enhanced nanoparticle accumulation in glioblastoma tissue in vivo. The OMIs encapsulated in SNA-gold liposome-ApoE were protected from degradation by serum components, indicating higher payload delivery to glioblastoma even in the presence of high protein and nuclease concentrations found in plasma [123].

## 7 Exosomes as vectors to carry miRNA agents

Exosomes, natural carriers of macromolecules and information between cells, have gained significant attention in the field of medicine and therapeutics. Unlike artificial nanoparticles, exosomes have the advantage of prolonged circulation in the body, increasing their chances of reaching distant target cells. Researchers have been exploring the engineering of exosomes to deliver therapeutic agents and nucleic acids to specific cells, particularly cancer cells.

In one study, exosomes were designed as in vivo vectors for targeted delivery of miRNAs to tumor cells [124]. These miRNAs play essential roles in regulating gene expression and have been recognized as potential therapeutic agents. The study successfully loaded miR-317b-5p into exosomes, allowing for the specific and efficient delivery of this therapeutic miRNA to tumor cells. This delivery resulted in notable changes in tumor cell activities, including viability, proliferation, apoptosis, migration, and invasion. Furthermore, the study extended its findings to an in vivo setting, demonstrating the antitumor efficacy of miR-317b-5p-loaded engineered exosomes in mice with osteosarcoma. Mice treated with these exosomes exhibited the smallest tumor volumes and the longest survival times, indicating significant antitumor effects compared to control groups. Importantly, miR-317b-5p-loaded engineered exosomes effectively delivered the therapeutic miRNA to tumor tissues when administered systemically. While this research shows promise for the use of engineered exosomes as nanocarriers for therapeutic molecules, it also highlights certain challenges. The manufacturing of engineered exosomes is complex and costly, and each formulation requires thorough molecular and functional characterization before clinical use. Despite these challenges, the results suggest that miR-317b-5p-loaded engineered exosomes have potential as effective anticancer agents, with the possibility of future development into injectable therapeutic drugs.

In another study, researchers investigated the use of mesenchymal stem cells (MSCs) as natural biofactories for the production of exosomes containing high levels of miR-124a [125]. MiR-124a has shown efficacy in inhibiting the growth of glioblastoma stem cells (GSCs), making it a promising anti-glioma agent. The study demonstrated that MSCs engineered to overexpress miR-124a effectively inhibited the growth of GSCs. Notably, the researchers introduced a novel approach by using exosomes as delivery vehicles for miR-124a. These exosomes, called Exo-miR124 exosomes, were shown to inhibit the survival and clonogenicity of GSCs in in vitro assays. Moreover, when administered systemically to mice with intracranial GSC xenografts, Exo-miR124 exosomes demonstrated the ability to cure the mice. The study suggests that MSCs can serve as ex vivo biofactories for producing exosomes loaded with anti-glioma miRNAs, such as miR-124a. Unlike MSCs, exosomes can be administered intravenously, allowing for easier and more frequent delivery. This approach combines an effective therapy (miR-124) with an efficient delivery mechanism (exosomes), offering a translational and clinically feasible strategy. Additionally, the study shed light on a potential mechanism underlying the effects of miR-124a on GSCs. It is known that some cancers, including glioblastoma, rely on capturing and metabolizing exogenous lipids for their growth. MiR-124a was found to downregulate forkhead box protein A2 (FOXA2), an oncogenic transcription factor associated with lipid metabolism. This downregulation resulted in reduced GSC viability and intracellular lipid accumulation, suggesting that miR-124a may hinder GSCs' ability to metabolize lipids.

In conclusion, these studies highlight the promising role of exosomes in the delivery of therapeutic molecules, particularly miRNAs, for cancer treatment. Engineered exosomes and MSCs as biofactories offer innovative approaches to target and inhibit tumor cells effectively. While challenges exist in terms of manufacturing and characterization, these findings provide a foundation for the development of novel therapies against various cancers, including glioblastoma. The potential clinical applications of these approaches could have a significant impact on cancer treatment strategies in the future.

## 8 MiRNAs and their contribution in neuro-oncology

The dysregulation of miRNAs in brain tumors offers an exciting opportunity for therapeutic intervention. Strategies for targeting miRNAs include miRNA mimics and inhibitors, as well as innovative approaches such as nanoparticle-based delivery. These emerging therapeutic avenues hold great promise for advancing the treatment of neuro-oncological diseases. One of the most promising avenues for miRNA-based therapies in neuro-oncology involves the use of miRNA mimics and inhibitors. These synthetic RNA molecules are specifically designed to either mimic the function of tumor suppressor miRNAs or block the activity of oncomiRs, which are often overexpressed in cancer cells [126,127].

One of the major challenges in the development of miRNA-based therapies for neuro-oncology is the effective delivery of these molecules to the CNS. The BBB poses a formidable obstacle, limiting the passage of therapeutic agents from the bloodstream into the brain. However, recent advancements in nanoparticle-based delivery systems offer a promising solution to this challenge. Nanoparticles are tiny structures, often in the nanometer range, that can encapsulate therapeutic molecules like miRNA mimics or inhibitors. These nanoparticles can be engineered to have specific properties that enable them to bypass or traverse the BBB [128]. Various nanoparticle formulations, including liposomes and polymeric nanoparticles, have been developed to facilitate the targeted delivery of miRNA-based therapies to brain tumors. Nanoparticles can be surface-modified with ligands or antibodies that recognize receptors or markers specific to cancer cells in the CNS. This targeted approach enhances the specificity of miRNA delivery, reducing potential off-target effects and maximizing therapeutic efficacy. Additionally, nanoparticles can provide controlled release of miRNAs over time, ensuring a sustained therapeutic effect. The use of nanoparticles for miRNA delivery not only addresses the challenge of crossing the BBB but also enhances the stability and bioavailability of miRNAs. This is crucial for achieving therapeutic concentrations of miRNAs in the tumor microenvironment. As a result, nanoparticle-based delivery approaches hold great potential for improving the overall efficacy of miRNA-based therapies in neuro-oncology [129].

In summary, the field of neuro-oncology is rapidly evolving, and miRNAs have emerged as pivotal players with the potential to revolutionize treatment strategies. MiRNA mimics and inhibitors offer a precise and targeted approach to modulating gene expression in cancer cells, while nanoparticle-based delivery systems address the challenges of delivering therapeutic agents to the CNS. As ongoing clinical trials continue to investigate the safety and efficacy of these innovative approaches, there is growing optimism that miRNA-based therapies will play a significant role in the future of neuro-oncological treatment [3,130].

The intricate web of genetic and epigenetic factors underlying neuro-oncological diseases has long posed significant challenges to clinicians and researchers alike. However, the discovery of the critical roles played by miRNAs in these complex processes has opened exciting new possibilities for diagnosis and treatment. MiRNAs, with their ability to fine-tune gene expression, have been shown to function both as tumor suppressors and oncogenes in neuro-oncology, influencing critical signaling pathways that drive cancer development. Moreover, their stability and accessibility in various bodily fluids make them valuable diagnostic and prognostic biomarkers, aiding in more accurate disease classification and treatment planning. The development of miRNA-based therapeutics, including miRNA-vector-based delivery systems, represents a promising frontier in neuro-oncological research. Clinical trials are underway to evaluate the safety and efficacy of these approaches, offering hope for more effective and targeted treatments for patients with neuro-oncological diseases. As our understanding of miRNAs continues to deepen, we can anticipate the discovery of new miRNA targets and the development of innovative therapies that may ultimately transform the landscape of neuro-oncology. This ongoing progress holds the promise of improved outcomes and increased hope for patients and clinicians alike, reinforcing the importance of continued research in this rapidly evolving field.

## 9 Conclusion

This research aimed to provide the most current insights into the existing methods for delivering siRNA agents, offering a promising and secure avenue for brain tumor treatment. Additionally, it sought to illuminate the challenges that impede the integration of these technologies into clinical neuro-oncology. It is worth noting that beyond aggressive glial tumors, other forms of brain malignancies, such as malignant meningiomas, medulloblastomas, and metastatic brain tumors, also demand extensive research and solutions for critical treatment issues. Consequently, the scope of these studies remains somewhat limited.

The potential of miRNA-based therapy technology is substantial in the realm of brain tumor treatment, as it enables precise modulation of the expression levels of oncogenes and tumor suppressors. However, the advancement of this therapeutic approach hinges on the development of secure and efficient carriers for the systemic delivery of miRNAs. Currently, non-viral vector systems exhibit lower transfection efficiency when compared to viral vectors. Hence, continuous efforts are necessary to bolster effectiveness and diminish the toxicity of non-viral delivery systems.

## Author contributions

Ilgiz Gareev: conceptualization, writing – original draft, and project administration. Ozal Beylerli: writing – review and editing and investigation. Tatiana Ilyasova, Rasim Tamrazov and Alina Shumadalova: formal analysis and methodology. Weijie Du and Baofeng Yang: resources and data curation, validation, and visualization. Baofeng Yang: supervision and funding acquisition. All authors have read and agreed to the published version of the manuscript.

## Funding

This work was supported by the 10.13039/501100001809National Natural Science Foundation of China (8227392, 8227392, U21A20339) and by the Bashkir State Medical University Strategic Academic Leadership Program (PRIORITY-2030).

## Patient consent for publication

Not applicable.

## Ethics approval and consent to participate

Not applicable.

## Availability of data and materials

Not applicable.

## Publisher's note

All claims expressed in this article are solely those of the authors and do not necessarily represent those of their affiliated organizations, or those of the publisher, the editors, and the reviewers. Any product that may be evaluated in this article, or claim that may be made by its manufacturer, is not guaranteed or endorsed by the publisher.

## Declaration of competing interest

The authors declare that the research was conducted in the absence of any commercial or financial relationships that could be construed as a potential conflict of interest.
